# Radioactive iodine refractoriness in Middle Eastern differentiated thyroid cancer: clinical outcome and risk factor analysis

**DOI:** 10.3389/fendo.2024.1326976

**Published:** 2024-05-15

**Authors:** Sandeep Kumar Parvathareddy, Abdul K. Siraj, Nabil Siraj, Saeeda O. Ahmed, Maha Al-Rasheed, Zeeshan Qadri, Khawar Siddiqui, Saif S. Al-Sobhi, Fouad Al-Dayel, Khawla S. Al-Kuraya

**Affiliations:** ^1^ Human Cancer Genomic Research, Research Center, King Faisal Specialist Hospital and Research Centre, Riyadh, Saudi Arabia; ^2^ Department of Pediatric Hematology-oncology, King Faisal Specialist Hospital and Research Centre, Riyadh, Saudi Arabia; ^3^ Department of Surgery, King Faisal Specialist Hospital and Research Centre, Riyadh, Saudi Arabia; ^4^ Department of Pathology, King Faisal Specialist Hospital and Research Centre, Riyadh, Saudi Arabia

**Keywords:** differentiated thyroid cancer, radioactive iodine refractory, risk factors, TERT mutation, DTC-specific survival

## Abstract

**Background:**

Radioactive iodine refractory differentiated thyroid cancer (RAIR-DTC) has received increasing attention due to its poor prognosis. However, outcomes may vary among patients with RAIR-DTC. The role of clinico-pathological and molecular prognostic factors in survival remains controversial, resulting in difficulty in selecting patients for new targeted therapies. We assessed mortality rate and DTC-specific survival in Middle Eastern RAIR-DTC to identify prognostic factors associated with survival.

**Methods:**

This single center, retrospective study enrolled 268 patients with RAIR-DTC. Mortality rate and DTC-specific survival were analyzed to identify prognostic factors related to survival. Univariate and multivariate analysis were performed using Cox proportional hazards model.

**Results:**

Of the 268 cases of RAIR-DTC, 40.3% (108/268) had absent 131I uptake (either on diagnostic or post-therapy whole body scan), 15.3% (41/268) had progressive disease (PD) despite ^131^I, 7.5% (20/268) had persistent disease despite cumulative activity of I^131^ of >600 mCi and 36.9% (n=99/268) developed distant metastasis. On multivariate analysis, age (more than 45 years), presence of metastatic disease and tumors harboring *telomerase reverse transcriptase* (*TERT*) promoter mutations were independent prognostic factors for poor DTC-specific survival. Subjects were divided into 3 groups according to the number of risk factors; low risk (no risk factors); intermediate (≤ 2 risk factors); and high risk (all the 3 risk factors). Ten-year DTC-specific survival rates in low, intermediate and high-risk groups were 100.0%, 92.9% and 53.6%, respectively.

**Conclusions:**

The contribution of age greater than 45 years to RAIR-DTC mortality is impactful. Older age, presence of distant metastasis and *TERT* mutations could be used as early predictors of RAIR-DTC cases. The identification of prognostic factors for poor survival in RAIR-DTC may improve the selection of patients for more personalized surveillance and therapeutic modalities.

## 1 Introduction

Recently, radioactive iodine refractory differentiated thyroid cancer (RAIR-DTC) has imposed a significant challenge as a result of increasing number of patients with DTC around the world (1). DTC, including Papillary Thyroid Carcinoma (PTC), Follicular Thyroid carcinoma (FTC), and Hurthle Cell Carcinoma (HCC), accounts for about 90% of all thyroid cancer (2–4). Most DTC patients can be treated successfully by surgery and radioactive iodine (RAI) with favorable outcome. Despite the favorable prognosis, recurrence and distant metastases occur in 2 – 30% of DTCs (5–7). Among these patients, unfortunately, a significant number show loss of iodine uptake (8). The efficacy of RAI therapy is largely influenced by the ability of tumors to take up radioiodine (9–11). A long-term study showed that 10- and 15-years survival rate in RAIR-DTC were much lower than those of DTC patients with RAI uptake (10% vs 56% and 6 vs 45%, respectively) (9). RAIR patients represent a great therapeutic challenge due to the limited alternative therapeutic options (12, 13). Therefore, understanding non-radioiodine avidity and identification of risk factor that help in early prediction of RAIR-DTC is of great clinical importance in avoiding unnecessary RAI therapy and help in the decision of subsequent feasible targeted therapy.

Patients’ age and other clinico-pathological as well as molecular risk factors for RAIR DTC have been explored in several studies with controversial results (14–18). Furthermore, the clinico-pathological associations, molecular features and prognostic impact of RAIR-DTC in Middle Eastern ethnicity has not been clarified. Therefore, we conducted this retrospective study to identify risk factors affecting DTC-specific survival in RAIR disease and risk stratification was also attempted based on the identified risk factors.

## 2 Materials and methods

### 2.1 Clinical cohort

Two-hundred and sixty eight RAIR DTC patients diagnosed between 1988 and 2018 at King Faisal Specialist Hospital and Research Centre (Riyadh, Saudi Arabia) were included in the study. The main inclusion criteria were histology of DTC (papillary or follicular cancer) and disease classified as RAIR after at least one dose of 131I treatment. The Institutional Review Board of the hospital approved this study and since only retrospective patient data were used, the Research Advisory Council (RAC) provided waiver of consent under project RAC # 221 1168 and # 2110 031. The study was conducted in accordance with the Declaration of Helsinki.

### 2.2 Definition of radioactive iodine refractoriness

Based on the recently published joint consensus from the American Thyroid Association, the European Association of Nuclear Medicine, the European Thyroid Association, the Society of Nuclear Medicine and Molecular Imaging on Current Diagnostic and Theranostic Approaches, and current literature (18–21), DTCs were classified as RAI refractory, if any of the following were fulfilled:

1. 131I uptake absent on diagnostic 131I scan of locoregional recurrence or distant metastasis.
2. 131I uptake absent on 131I scan, performed several days after 131I treatment.
3. 131I uptake present in some, but not all tumor foci.
4. Disease progression despite a cumulative 131I activity of ≥600mCi.
5. Metastatic disease progression despite 131I uptake.
6. Rising serum thyroglobulin levels ≥6 months after 131I treatment.
7. Structural disease progression after 131I treatment.

### 2.3 Clinico-pathological and follow-up data

Baseline clinico-pathological data were collected from case records and have been summarized in **Table 1**. Staging of DTC was performed using the eighth edition of American Joint Committee on Cancer (AJCC) staging system (22). The patients were seen 6 to 8 weeks after surgery, having been prepared with thyroid hormone withdrawal for at least four weeks and low-iodine diet for one week, in order to achieve a target TSH level of >30µIU/mL. A diagnostic radioactive iodine (I-123) whole body scan (DxWBS) and neck ultrasonography were performed, and stimulated thyroglobulin (sTg), anti-Tg antibodies, TSH and free T4 were measured. Radioactive iodine (I-131) was administered at activities that averaged 30–100 mCi for thyroid remnant ablation and 100–200 mCi for patients with lymph node or distant metastases. The study endpoint for our analysis was DTC-specific survival, defined as the time (in years) from date of initial surgery to the date of death due to progression of DTC.

**Table 1:** Patient characteristics of the radioactive iodine refractory differentiated thyroid cancer (RAIR-DTC).

	Overall cohort (n = 268)	
Age at RAIR diagnosis (years)		
Median (range)	44.6 (13.0 – 95.0)	
Gender		
Male	90	33.6
Female	178	66.4
Histologic subtype		
Papillary thyroid cancer (PTC)	253	94.4
Follicular thyroid cancer (FTC)	15	5.6
Extrathyroidal extension		
Present	164	61.2
Absent	104	38.8
Lymphovascular invasion		
Present	75	28.0
Absent	193	72.0
Tumor focality		
Unifocal	127	47.4
Multifocal	141	52.6
Tumor laterality		
Unilateral	163	60.8
Bilateral	105	39.2
Tumor size (cm)		
Mean (± SD)	3.6 (± 2.2)	
Lymph node metastasis		
Absent	72	28.7
Present	179	71.3
Distant metastasis		
Absent	169	63.1
Present	99	36.9
TNM Stage		
I	186	69.4
II	47	17.5
III	5	1.9
IV	30	11.2
*BRAF* mutation		
Present	149	57.3
Absent	111	42.7
*TERT* mutation		
Present	68	26.3
Absent	191	73.7
RAIR categories		
Absent 131I uptake on DxWBS	67	25.0
Absent 131I uptake on post-therapy WBS	26	9.7
131I uptake present in some, but not all tumor foci	15	5.6
Disease progression despite a cumulative 131I activity of ≥600mCi	20	7.5
Metastatic disease progression despite 131I uptake	29	10.8
Rising serum thyroglobulin levels ≥6 months after 131I treatment	41	15.3
Structural disease progression after 131I treatment	70	26.1

### 2.4 DNA isolation

DNAs were extracted from PTC formalin-fixed and paraffin-embedded (FFPE) tumor tissues utilizing Gentra DNA isolation kit (Gentra, Minneapolis, MN, USA) according to manufacturer’s protocols as elaborated in the previous studies (23).

### 2.5 Sanger sequencing analysis

PCR and Sanger sequencing analysis of the promoter region in *TERT* gene were carried out as described previously (24). Primer 3 online software was utilized to design the primers (available upon request). Reference sequences were downloaded from NCBI GenBank. Sequencing results were compared with the reference sequence by Mutation Surveyor V4.04 (Soft Genetics, LLC, State College, PA).

### 2.6 Statistical analysis

DTC-specific survival rates were calculated by the Kaplan-Meier method. Cox proportional hazards model was used for analyzing the impact of prognostic factors on DTC-specific survival in univariate and multivariate manner. Risk stratification was performed according to the factors related to survival. Limit of significance was defined as p value < 0.05. Data analyses were performed using the JMP14.0 (SAS Institute, Inc., Cary, NC) software package.

## 3 Results

### 3.1 Patient and tumor characteristics

Median age at RAIR diagnosis for the entire cohort was 44.6 years (range = 13 – 95 years), with a male: female ratio of 1:2. Majority of the tumors were PTC (94.4%; 253/268). Extrathyroidal extension was noted in 61.2% (164/268) of cases and lymphovascular invasion in 28.0% (75/268). 52.6% (141/268) of PTCs were multifocal and 39.2% (105/268) were bilateral. Lymph node metastasis was noted in 71.3% (179/251) cases. BRAF mutation analysis was performed in 260 cases and TERT mutation analysis in 259 cases, with mutations noted in 57.3% (149/260) and 26.3% (68/259) cases, respectively (**Table 1**).

### 3.2 Association between patient age and DTC-specific mortality in RAIR patients

**Figure 1:**
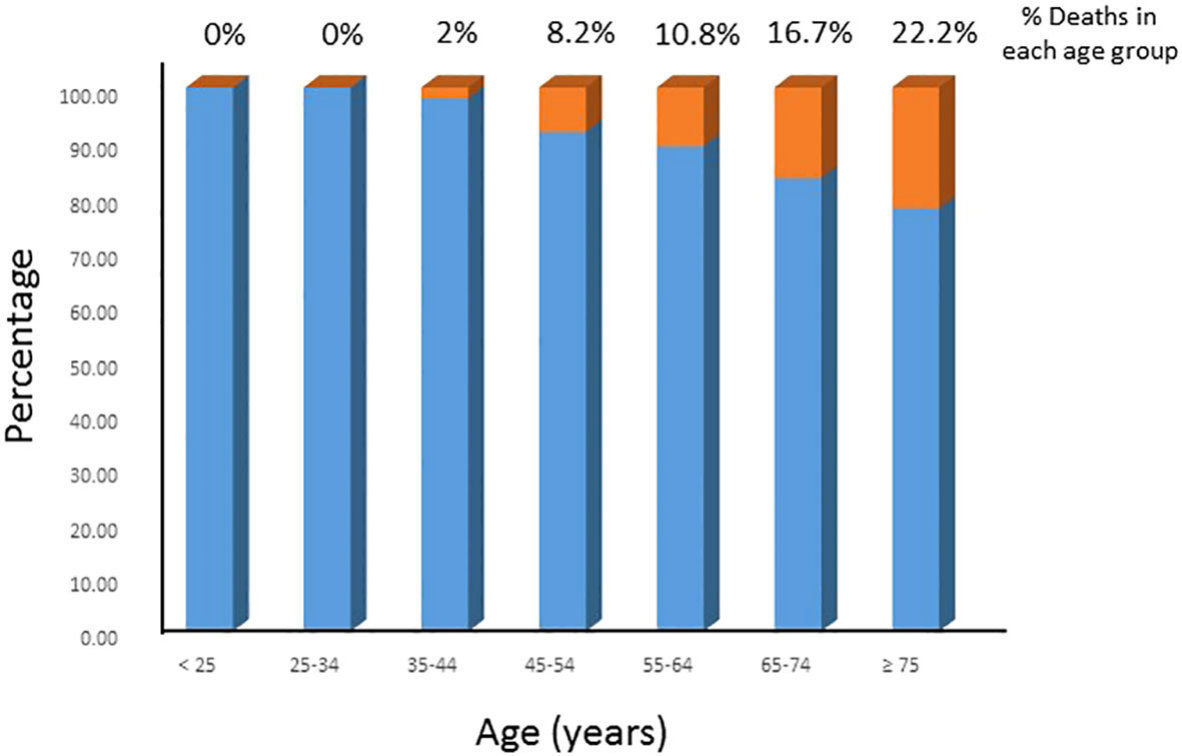
DTC-specific mortality rate in different age groups. The DTC-specific mortality rates increased with increasing age in the entire cohort.

**Figure 2:**
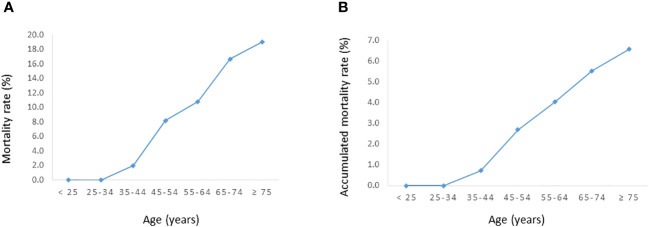
Relationship between age and DTC-specific mortality rates in the entire cohort. **(A)** Mortality rate. **(B)** Accumulated mortality rate.

The DTC-specific mortality rate in the entire cohort was 6.7% (18/268). **Figure 1** shows the DTC-specific mortality rate for different age groups. As shown in **Figure 2**, before the age of 45 years, the mortality rates (percentages of deaths in the cohort) were low in the entire cohort. After the age of 45 years, mortality rates increased as patient age increased in all patients (**Figure 2A**). Accumulated mortality rates also increased continuously after age 45 years in all patients (**Figure 2B**).

**Figure 3:**
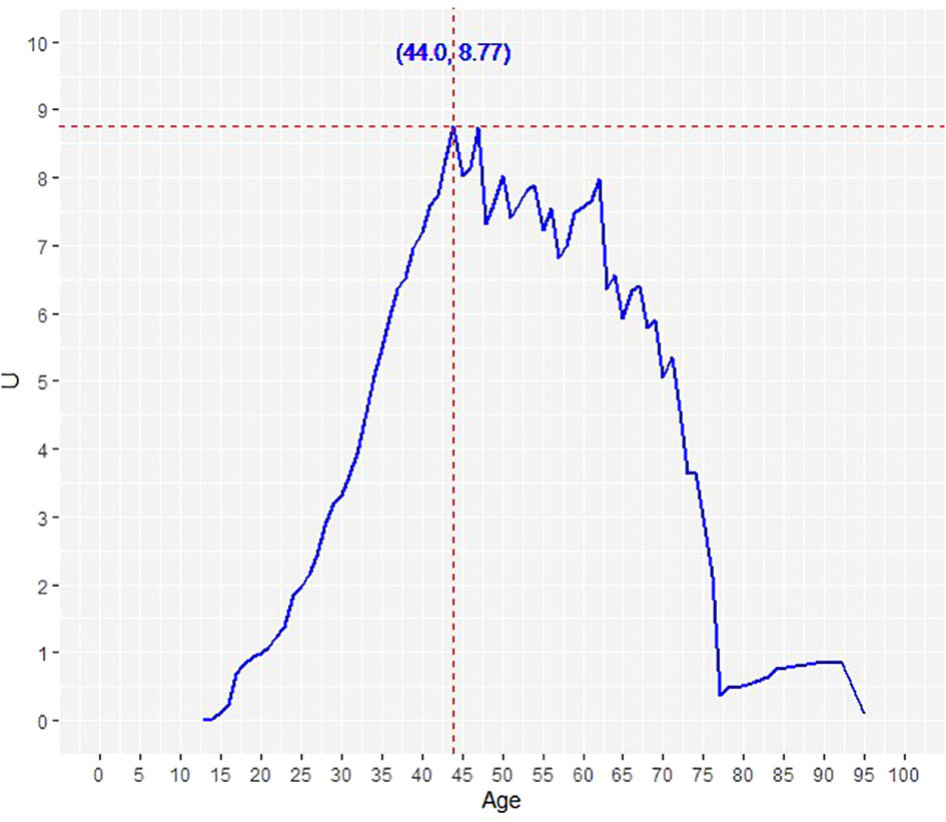
Determination of the optimal age cut-off point for DTC-specific survival using Contal and O’Quigley’s method. The dashed line demarcates the optimal age cut-off point: 44.0 years.

We further sought to determine the optimal age cutoff for predicting DTC-specific mortality. Using the Contal and O’Quigley method for DTC-specific mortality, the optimal age cutoff was determined to be 44 years (**Figure 3**). Based on the above findings and since the median age of our cohort was also close to 45 years, we used the age cut-off of 45 years for subsequent univariate and multivariate analysis.

### 3.3 Risk factors for DTC-specific mortality and risk stratification

**Figure 4:**
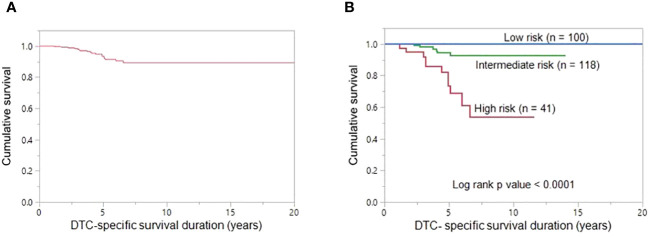
DTC-specific survival. **(A)** The 5- and 10-year DTC-specific survival rates in the entire cohort (n = 268) were 93.4% and 89.3%, respectively. **(B)** The 10-year DTC-specific survival rates in the low-, intermediate-, and high-risk groups are 100.0%, 92.9% and 53.6%, respectively. DTC-specific survival is significantly better in low-risk group patients than in high-risk group patients and intermediate-risk group patients (p < 0.0001).

The 5- and 10-year DTC-specific survival rates for the entire cohort were 93.4% and 89.3%, respectively (**Figure 4A**). On univariate analysis, age ≥45 years (p < 0.0001), tumor size (p = 0.0052), distant metastasis (p < 0.0001) and *TERT* mutation (p < 0.0001) were significantly related to DTC-specific survival (**Table 2**). However, on multivariant analysis, age ≥45 years (Hazard ratio (HR) = 7.41; 95% confidence interval (CI) = 1.20 – 142.58; p = 0.0295), distant metastasis (HR = 3.43; 95% CI = 1.31 – 10.74; p = 0.0110) and *TERT* mutation (HR = 3.96; 95% CI = 1.53 – 12.14; p = 0.0036) were found to be independent predictive markers of poor DTC-specific survival in this cohort (**Table 2**).

**Table 2:** Cox proportional hazards model for predictors of DTC-specific survival in RAIR-DTC.

	DTC-specific survival			
	Univariate		Multivariate	
Clinico-pathological variables	HR (95% CI)	p value	HR (95% CI)	p value
Age at RAIR diagnosis				
≥ 45 years (vs. < 45 years)	27.90 (5.90 – 498.56)	< 0.0001	7.41 (1.20 – 142.58)	0.0295
Gender				
Male (vs. Female)	0.89 (0.36 – 1.98)	0.7793		
Histologic subtype				
PTC (vs. FTC)	0.52 (0.15 – 3.25)	0.4186		
Extrathyroidal extension				
Present (vs. absent)	1.02 (0.47 – 2.33)	0.9603		
Lymphovascular invasion				
Present (vs. Absent)	1.80 (0.79 – 3.93)	0.1573		
Tumor focality				
Multifocal (vs. unifocal)	0.93 (0.43 – 2.05)	0.8578		
Tumor laterality				
Bilateral (vs. Unilateral)	1.19 (0.54 – 2.56)	0.6618		
**Tumor size (per unit change)**	1.23 (1.06 – 1.41)	0.0052	1.80 (0.26 – 11.58)	0.5476
Lymph node metastasis				
Present (vs. absent)	0.92 (0.40 – 2.39)	0.8593		
Distant metastasis				
Present (vs. absent)	6.76 (2.87 – 18.50)	< 0.0001	3.43 (1.31 – 10.74)	0.0110
***Stimulated Tg (per unit change)***	1.00 (0.99 – 1.01)	0.2876		
*BRAF* mutation				
Present (vs. absent)	0.84 (0.39 – 1.85)	0.6562		
*TERT* mutation				
Present (vs. absent)	12.27 (5.11 – 34.11)	< 0.0001	3.96 (1.53 – 12.14)	0.0036

HR, Hazard ratio; CI, Confidence interval; PTC, Papillary thyroid cancer; FTC, Follicular thyroid cancer; Tg, Thyroglobulin.

Based on the number of independent risk factors, patients were divided into 3 groups: low risk (no risk factors); intermediate risk (≤ two risk factors); and high risk (all three risk factors). Risk stratification was performed for 259 patients for whom *TERT* mutation data was available. 38.6% (100/259), 45.6% (118/259) and 15.8% (41/259) of patients were classified as low-, intermediate- and high-risk, respectively. 10-year DTC-specific survival rates in the low, intermediate and high risk groups were 100.0%, 92.9% and 53.6%, respectively (p < 0.0001) (**Figure 4B**).

## 4 Discussion

Recently, RAIR-DTC has received increasing attention due to its impact on patient survival. Better understanding of early predictors of RAIR-DTC is of great clinical importance to prevent unnecessary repeated use of radioactive iodine therapy and help physicians in tailoring patients’ surveillance and exploring other alternative modalities.

Age is a well-established prognosticator and mortality risk factor in general (25–27). Upon exploring the relationship between patient age and disease-specific survival in DTC, emerging data have appeared to question the appropriateness of dichotomizing age, given the inconsistency of published data on this subject (25–31). A recent study has analyzed the influence of age on survival of patients with RAIR-DTC and identified cutoff age of 45 years as being predictive of overall survival (14).

Our current study explored the relationship between age and DTC-specific mortality in RAIR-DTC. In unadjusted analysis, increasing patient age was associated with progressive increase in mortality. Before age of 45 years, the mortality rates were low in all age groups. However, after the age of 45 years, mortality rate and accumulated mortality rate increased continuously in all patients. We used a cutoff age of 45, which is close to the median age of our cohort. Furthermore, using statistical adjustments between young and older group, we attempted to establish the cutoff point for DTC-specific survival using Contal and O’Quigley’s method. Interestingly, the statistically suggested cutoff point for DTC-specific survival was 44 years, which further supports our suggested cutoff point of 45 years. Using this value, we performed multivariate analysis to identify clinico-pathological and molecular risk factors for reduced DTC-specific survival.

In this series, we identified older age (> 45 years), presence of distant metastasis and *TERT* promoter mutations as independent predictors for poor DTC-specific survival. Previous studies have shown the correlation between older age and poor patient outcome in RAIR-DTC (14, 32). Aggressive tumors, especially the presence of metastasis, is a useful feature for indicating worse prognosis in DTC (33, 34). *TERT* promoter mutation has been reported by us and other groups to be associated with aggressive clinico-pathological characteristics, including its association with RAIR-DTC (35–37). A recent study examined the status of *TERT* mutation in distant metastatic DTC and its association with RAI uptake as well as therapy response, and identified *TERT* mutation as having a greater negative influence on RAI uptake compared to *BRAF* mutation (38).

According to the number of significant risk factors related to poor DTC-specific survival, we attempted to perform risk stratification; low risk group were defined as patients having no risk factor, intermediate risk as patients having one or two risk factors and patients having all three risk factors combined were classified as high risk. Interestingly, based on the risk stratification, there was a significant difference in mortality between low-, intermediate- and high-risk groups, with the 10-year DTC-specific survival rates being 100.0%, 92.9% and 53.6%, respectively.

Due to the limited number of patients, our risk stratification should be interpreted with caution. However, risk adapted management of RAIR-DTC should be explored. Limitations caused by retrospective design of our study and modification to RAIR-DTC management over extensive follow-up period of more than 20 years cannot be ruled out. A prospective well-standardized study may help in providing more accurate information. In conclusion, older age, distant metastasis and *TERT* mutation are independent predictors of RAIR-DTC in Middle Eastern ethnicity. These markers could improve assessment of prognosis in RAIR-DTC patients and thus help clinicians in the selection of optimum therapeutic modalities, such as aggressive treatment and follow-up for those with worse DTC-specific survival.

## Data availability statement

The original contributions presented in the study are included in the article. Further inquiries can be directed to the corresponding author.

## Ethics statement

The studies involving humans were approved by Research Advisory Council, King Faisal Specialist Hospital and Research Centre. The studies were conducted in accordance with the local legislation and institutional requirements. The human samples used in this study were acquired from a by- product of routine care or industry. Written informed consent for participation was not required from the participants or the participants’ legal guardians/next of kin in accordance with the national legislation and institutional requirements.

## Author contributions

SP: Conceptualization, Data curation, Investigation, Validation, Writing – review & editing. AS: Conceptualization, Investigation, Validation, Visualization, Writing – original draft, Writing – review & editing. NS: Investigation, Methodology, Writing – review & editing. SA: Data curation, Investigation, Methodology, Writing – review & editing. MA: Investigation, Methodology, Writing – review & editing. ZQ: Formal Analysis, Methodology, Software, Writing – review & editing. KS: Formal Analysis, Software, Writing – review & editing. SA: Resources, Writing – review & editing. FA: Resources, Writing – review & editing. KA: Conceptualization, Project administration, Supervision, Visualization, Writing – original draft, Writing – review & editing.
